# Organic matter reduces the amount of detectable environmental DNA in freshwater

**DOI:** 10.1002/ece3.6123

**Published:** 2020-03-21

**Authors:** Kees van Bochove, Freek T. Bakker, Kevin K. Beentjes, Lia Hemerik, Rutger A. Vos, Barbara Gravendeel

**Affiliations:** ^1^ Biosystematics Group Wageningen University Wageningen The Netherlands; ^2^ Naturalis Biodiversity Center Leiden The Netherlands; ^3^ Institute of Biology Leiden Leiden University Leiden The Netherlands; ^4^ Biometris Wageningen University Wageningen The Netherlands; ^5^ Institute for Water and Wetland Research Radboud University Nijmegen The Netherlands

**Keywords:** degradation, detection, environmental DNA, *Gammarus pulex*, organic matter, pH

## Abstract

Environmental DNA (eDNA) is used for monitoring the occurrence of freshwater organisms. Various studies show a relation between the amount of eDNA detected and target organism abundance, thus providing a potential proxy for reconstructing population densities. However, environmental factors such as water temperature and microbial activity are known to affect the amount of eDNA present as well. In this study, we use controlled aquarium experiments using *Gammarus pulex* L. (Amphipoda) to investigate the relationship between the amount of detectable eDNA through time, pH, and levels of organic material. We found eDNA to degrade faster when organic material was added to the aquarium water, but that pH had no significant effect. We infer that eDNA contained inside cells and mitochondria is extra resilient against degradation, though this may not reflect actual presence of target species. These results indicate that, although estimation of population density might be possible using eDNA, measured eDNA concentration could, in the future, be corrected for local environmental conditions in order to ensure accurate comparisons.

## Graphical abstract

We found eDNA to degrade faster when organic material was added to the aquarium water, but that pH had no significant effect. Results indicate that, although estimation of population density might be possible using eDNA, measured eDNA concentration could, in the future, be corrected for local environmental conditions in order to ensure accurate comparisons.

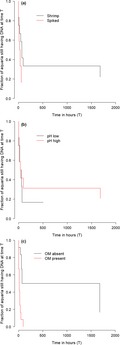

## 1 INTRODUCTION

DNA extracted from the environment is referred to as environmental DNA (eDNA), which is usually degraded (Taberlet, Coissac, Hajibabaei, & Rieseberg, 2012; Taberlet, Bonin, Coissac, & Zinger, 2018). Environmental DNA that is extracted from freshwater samples may originate from feces, urine, skin, and excreted tissue and can be free, cellular or particle‐bound (e.g., Levy‐Booth et al., 2007; Pietramellara et al., 2009). Although it is often highly degraded, it is possible to PCR amplify small fragments of eDNA such that even species that occur at low abundances can be detected from, for instance, water samples (Dejean et al., 2011, 2012; Jerde, Mahon, Chadderton, & Lodge, 2011). Among others, Katano, Harada, Doi, Souma, and Minamoto (2017) and Thomsen, Kielgast, Iversen, Wiuf, et al. (2012) have shown that eDNA can, therefore, be used to quantitatively monitor the occurrence of various freshwater organisms.

Physical, chemical, and biological degradation, for example, by DNases and microbial activity, is known to compromise amplification (Levy‐Booth et al., 2007; Shapiro, 2008). Several studies show that under controlled conditions eDNA in aquatic environments is degraded beyond detectability within a week (Dejean et al., 2011; Eichmiller, Best, & Sorensen, 2016; Takahara, Minamoto, Yamanaka, Doi, & Kawabata, 2012; Thomsen, Kielgast, Iversen, Møller, et al., 2012; Thomsen, Kielgast, Iversen, Wiuf, et al., 2012) and that a positive relationship exists between the abundance of target organisms and eDNA concentration (Dejean et al., 2011; Takahara et al., 2012; Thomsen, Kielgast, Iversen, Wiuf, et al., 2012). Maruyama, Nakamura, Yamanaka, Kondoh, and Minamoto (2014) report degradation rates of fish eDNA in freshwater of up to 10% per hour and found a strong correlation with the developmental stage of the target organisms. The authors state that quantitative eDNA data from the field should, therefore, be corrected to control for postsampling degradation. To better understand the relationship between target organism abundance, field eDNA degradation rate, and developmental state, more data should be gathered on factors that influence degradation of and the ability to detect eDNA. Insight in the limits of eDNA detection is essential to prevent false negatives (Darling & Mahon, 2011). Known factors that affect DNA degradation are water temperature (e.g., Dupray, Caprais, Derrien, & Fach, 1997; Eichmiller et al., 2016; Lindahl, 1993; Palmer, Tsai, Paszko‐Kolva, Mayer, & Sangermano, 1993; Takahara et al., 2012; Tsuji, Ushio, Sakurai, Minamoto, & Yamanaka, 2017), UV level (Strickler, Fremier, & Goldberg, 2015), and DNA‐consuming microorganisms (Alvarez, Yumet, Santiago, & Toranzos, 1996; Dupray et al., 1997; Finkel & Kolter, 2001). Other factors that influence the rate of decay or the detectability of eDNA may be water conductivity and pH (Strickler et al., 2015; Thomsen, Kielgast, Iversen, Wiuf, et al., 2012), as well as the presence of organic matter (Saeki, Ihyo, Sakai, & Kunito, 2011).

Here, we study the effect of organic matter (hereafter referred to as OM) and pH on eDNA degradation and detection efficiency in an aquarium experiment using common freshwater shrimp (*Gammarus pulex* L., Amphipoda) as model species and DNA source. We hypothesize that both survival and accumulation of eDNA would be affected by pH and OM. Furthermore, we test whether extracellular DNA responds differently to pH and OM compared to eDNA released by dying shrimps. In addition, we test the level of PCR inhibition in all aquariums and whether it is affected by pH and OM.

## 2 MATERIALS AND METHOD

### 2.1 Environmental conditions

Our experimental design is summarized in Table 1 and encompassed eight treatments A–H. We filled each of 28 aquariums with 3.7 L water obtained from a natural water system in The Netherlands (GPS: N 52 10.056, E 4 28.086). We kept the aquariums under controlled conditions in the laboratory facility of Naturalis Biodiversity Center (Leiden, the Netherlands). There was no gravel or substrate inside, and the aquariums were not aerated. The aquariums were placed on a laboratory bench, and the treatments were equally distributed over the space. We varied the pH in the aquariums to either “high” (above 8) or “low” (below 5.7) and the amount of OM to either 10 g added, or none at all, resulting in four treatments (see Table 1). OM content of the water was increased in the following way: 5 g of decaying leaf material of locally growing plane trees (*Platanus hispanica*) and 5 g of leaf material of locally growing European beech (*Fagus sylvatica*) was added to the water after sterilizing the leaves for 1 hr at 120°C to prevent introduction of microorganisms that degrade eDNA. To lower the pH, we acidified the water using 3.7% HCl to a pH of 5. During the experiments, we monitored the pH of the water (17 measurements, Table S1) and we added additional HCl if the pH exceeded 5.7. We refilled the aquariums to the original level, each time that samples were collected for eDNA sampling of pH monitoring. The water in the aquariums was kept at room temperature.

**Table 1:** The various treatments and aquarium numbers

Treatment code	Aquarium no.	pH	OM	DNA source	Mean survival time (hr)
A	1, 11, 21	4–5.7	No	Shrimps	324.00
B	7, 17, 27	8–8.6	No	Shrimps	2,520.00
C	3, 13, 23	4–5.7	Added	Shrimps	24.67
D	5, 15, 25	8–8.6	Added	Shrimps	36.67
E	6, 16, 26	4–5.7	No	Spiked	67.00
F	2, 12, 22	8–8.6	No	Spiked	84.00
G	4, 14, 24	4–5.7	Added	Spiked	20.67
H	8, 18, 28	8–8.6	Added	Spiked	8.67

Note

The mean survival time (time to the disappearance of the DNA) is calculated as total time on test divided by the number of aquaria in which the DNA disappeared, meaning that it was at that time for the first time below the detection limit of 8,221 molecules per liter.

### 2.2 Inoculation of living shrimps

Prior to inoculation with DNA sources, we took samples from all 28 aquariums to estimate the level of background DNA of *Gammarus pulex* present. Four aquariums were not inoculated with any DNA source and served as control.

In 12 aquariums, we added eight live shrimps (*G. pulex*) in the final stages of their development. Last instars were chosen to avoid differences in molting and propagation between the aquariums. All individuals used in this study were collected in Wageningen, the Netherlands, from a single population in the wild (GPS: N 51 58.500, E 5 38.820). We removed dead shrimps and replaced them with live ones, and we also removed newborn shrimps (for details see supplemental material: Table S2).

### 2.3 Spiking DNA

On the date that we removed the shrimps from the aquariums, we spiked another twelve aquariums with 4.99 μg tissue‐derived extracellular genomic DNA of *G. pulex*. We measured DNA degradation in these aquariums from 2 hr after spiking, measuring every 60 min. The DNA used for spiking was extracted from tissue of *G. pulex* using the Qiagen DNeasy Blood & Tissue following the Spin‐column protocol. We measured DNA concentration in the extracts using a Qubit 2.0 fluorometer (Life Technologies).

### 2.4 Real‐time quantitative PCR

Environmental DNA degradation was monitored using a CFX96™ Real‐Time PCR System. We developed a species‐specific qPCR primer set using Geneious (PulexF1: ACGTAGACCTGGTATATCTATAGACC & PulexR1 CCGGCTAAAACAGGTAAGGA) to amplify a 98bp fragment of COI; we developed another primer set using primer‐BLAST of NCBI ((Ye et al., 2012) (PulexF2: GGAGCTTGGGCTAGTGTTGT and PulexR2: CGTGAGCGGTGACTAATGACG) to amplify an 118 bp fragment of COI. Both primer combinations worked well, but we selected primer pair PulexF1 & PulexR1 to do the experiment. We checked the specificity of both primers in silico using primer‐BLAST (2013/02/28) with the setting that unintended targets should have at least two mismatches within the last five base pairs at the 3′ end for one of the primers. Primer‐BLAST only showed hits of indigenous organisms except for *Gammarus duebeni*. However, in the case of the primer pair PulexF1 & PulexR1 a total of seven mismatches was found. Furthermore, *G. duebeni* does not occur in the region and occurs in a habitat type different from that at the location where we obtained aquarium water.

### 2.5 eDNA extraction

For extracting eDNA, we added 15 ml of water samples to 1.5 ml of 3 M sodium acetate and 33ml absolute ethanol and stored it at −20°C (following Ficetola, Miaud, Pompanon, and Taberlet (2008)). We centrifuged the mixture (9,400 ***g***, 35 min, 6°C) and discarded the supernatant. To extract DNA from the pellets, we used the Qiagen DNeasy Blood & Tissue kit (spin‐column protocol) after Thomsen, Kielgast, Iversen, Wiuf, et al. (2012); Thomsen, Kielgast, Iversen, Møller, et al. (2012). Quantitative real‐time PCR (qPCR) was performed in a total volume of 20 µl using 10 µl GoTaq PCR Master Mix 2X (Promega), 0.4 µl of both primers, 5.2 µl nuclease‐free water, and 4 µl template. We performed PCRs in 96‐well plates and included in each plate at least one negative and one positive PCR control reaction (both in triplicate).

### 2.6 eDNA sampling

We sampled eDNA in the aquariums 28 days after they had been inoculated with live shrimps to estimate the amount of eDNA that had been accumulated. Thereafter, the shrimps were removed. To estimate the survival of eDNA, samples were collected after 12, 24, 36, 48, 60, 72, 96, 168, 288, 504, 1,008, and 1,680 hr. We stopped sampling when the average C*_t_*‐value of a sample exceeded 47 (see below).

### 2.7 Avoiding false positives

In this study, we took several measures to avoid false positives (i.e., detecting eDNA when no animals were around). For detection of invertebrates in field samples, the use of specific‐binding probes is paramount for reliably detecting target organisms. Even when the concentration is extremely low, this approach can result in more sensitive and specific detection of target DNA (Goldberg et al., 2016; Schultz & Lance, 2015). However, because the concentration of eDNA in our controlled aquariums was relatively high we were able to use a less sensitive, low‐cost approach including GoTaq qPCR 2X Master Mix in a real‐time quantitative PCR assay, which contained BRYT Green, a fluorescent dye that binds to double‐stranded DNA. Since BRYT Green dye binds to all double‐stranded DNA, the presence of double‐stranded nontarget DNA, such as primer dimers, can also result in a fluorescent signal. Ct‐values were converted to numbers of molecules based on the principle that 2[Ct_Standard_ − Ct_Sample_] is the fold difference in concentration of sample and standard used. Standards (i.e., series of increasing known concentrations) were made for each PCR plate and resulting Ct‐values plotted against the 10log (number of molecules). Linear regression analysis of the average across plates then enabled calibrating the standards and calculating numbers of molecules in the aquarium samples. The detection limit was, thereafter, determined based on sample concentrations collected from control aquariums and from all other aquariums prior to inoculation (see also Figure S1).

### 2.8 Defining the amount of detectable eDNA

Each aquarium was sampled twice at each sampling time. Three water samples were collected from the aquariums with live shrimps just before they were removed from the aquariums, to be able to accurately determine the accumulation of eDNA in de aquariums. In 12 samples, the DNA pellet did not form properly during extraction, in which cases only one sample was analyzed.

### 2.9 Quantifying qPCR inhibition

We quantified the amount of PCR inhibition in the samples (*N* = 36) that were collected from the aquariums containing shrimps just before they were removed from aquariums. We did this by performing an inhibition qPCR test (see details below). We repeated this in the samples obtained from the spiked aquariums just after spiking (*N* = 23) and in the samples collected from the control aquariums that were obtained at the same time (*N* = 8). The qPCRs were spiked with an artificial fragment of DNA (CGGAGGTGCACTTACAGATAGAGTCACATGTCGTGTCTAACGCGCAGCAGTAGTGTCTGAACACGAGTCCTTCC) cloned into an pUC57 plasmid. The primers ART3‐*F* (CGGAGGTGCACTTACAGATAGAG) and ART3‐R (GGAAGGACTCGTGTTCAGACA) were used to amplify the fragment. For each sample, three qPCRs were performed containing 33, 333, and 3,333 molecules of the artificial DNA fragment.

We performed the inhibition qPCRs in a total volume of 20 µl using 10 µl GoTaq PCR Master Mix 2X (Promega), 0.4 µl of both primers, 4.2 µl nuclease‐free water, 4 µl template (either aquarium water or nuclease‐free distilled water), and 1 µl containing the artificial DNA molecules. The cycling conditions were identical to those used for detection of the shrimp DNA. A standard curve was generated using each DNA concentration in triplicate, in which nuclease‐free distilled water was added instead of aquarium sample. We assessed response variable Ct‐values of the control, shrimp and spiked data and explanatory variable Ct‐values of the standard deviated from slope 1 in order to assess the presence of inhibition. Therefore, linear mixed effect models were used (see below under Section 3.1). The *R* ^2^ and efficiency of the qPCR assay were calculated based on a standard containing 10, 100, 1,000, 10,000 target‐molecules (results not shown).

### 2.10 Statistics

In general, best‐fitting models were selected with Akaike's Information Criterion corrected for small sample sizes (AICc, see Equation 1):

(1) $$\text{AICc}=2k-2\log\left(L\right)+\left(2k\left(k+1\right)\right)/\left(n-k-1\right)$$

with log denoting the natural logarithm, *L* the likelihood of the model, *k* the number of estimated parameters in the model, and *n* the sample size (Bolker, 2008). The minimum AICc value indicates the best‐fitting model. Model fits are evaluated with respect to the AICc‐difference (ΔAICc) between the considered model and the best model. Models within the interval ΔAICc < 2 are considered equivalent (Bolker, 2008). In this set of models, Ockham's razor (parsimony criterion) was used to choose the best model, containing the smallest number of parameters. We used Fisher's least square difference (LSD) test with Bonferroni correction from the agricolae R library (de Mendiburu & de Mendiburu, 2017) to test for differences in the amount of eDNA accumulated in the aquariums with live shrimps.

## 3 RESULTS

### 3.1 Controls and limit of detection

The qPCR assay had typically a *R* ^2^ of over .99 and an efficiency of 70%. Although PCR efficiency was quite low, we could amplify even single molecules, indicating that the assay was rather sensitive. The negative PCR control reactions did not result in any amplification. However, we amplified low levels of eDNA from the samples collected prior to inoculation with DNA sources and in the samples collected from the control aquariums. For all samples analyzed of the control aquariums and samples collected prior to inoculation, we plotted a cumulative density function in R (R core team, 2014) on the Ct‐values. From this, we estimated the 5% percentile to be 45.5 (Figure S1). Based on this result, we estimated our detection limit to be 45 cycles, which given our standards corresponds to 8,221 molecules of DNA. Ct‐values exceeding 45 in the noncontrol measurements were subsequently set to 45 as such values are likely to be caused by low levels of contamination or by double‐stranded nontarget DNA, such as primer dimers. Based on the melting curve of the positive controls, the typical melting temperature of target DNA (e.g., the temperature that the highest amount of DNA products dissociates and becomes single‐stranded) was inferred to be in the range of 75.5 and 77.5°C. We assumed reactions showing a melting temperature outside this range to be nontarget DNA such as primer dimers. Therefore, we set their Ct‐values to 45.

### 3.2 eDNA accumulation in aquariums with live shrimps

**Figure 1:**
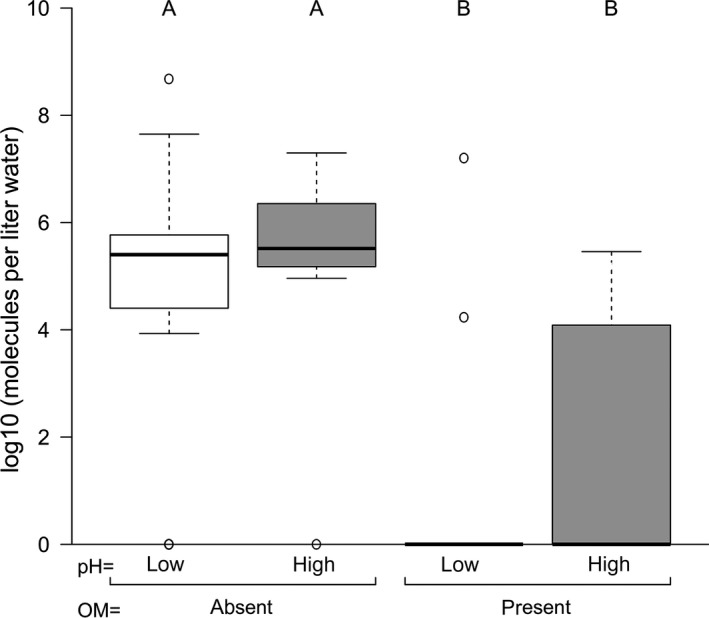
The relationship between number of DNA molecules present in water in experimental aquarium set‐ups under both high and low pH as well as presence/absence of organic matter (OM). DNA is of live freshwater shrimps after 28 days or spiked DNA 2 hr after spiking. The logarithmically transformed number of eDNA molecules per liter water is shown for the four experimental combinations: OM absent pH low, OM absent pH high, OM present pH low, and OM present pH high. The A and B in the figure denote the significantly different groups according to a LSD test at the 5% level. The bands near the middle of the boxplot show the median. The bold horizontal lines are drawn at the median values, whereas the box shows the interquartile range (IQR), which is the range between the 25% (Q1) point and 75% (Q3) point for the data. Therefore, 50% of the observations are in the interquartile range. The other half of the observations is at each side of the box (25% at either side). The whiskers extend to the most extreme data point, which is less than 1.5 times IQR of the box. Observations that are more than 1.5 IQR away from the nearest quartile (Q1 or Q3) are shown as circles

**Figure 2:**
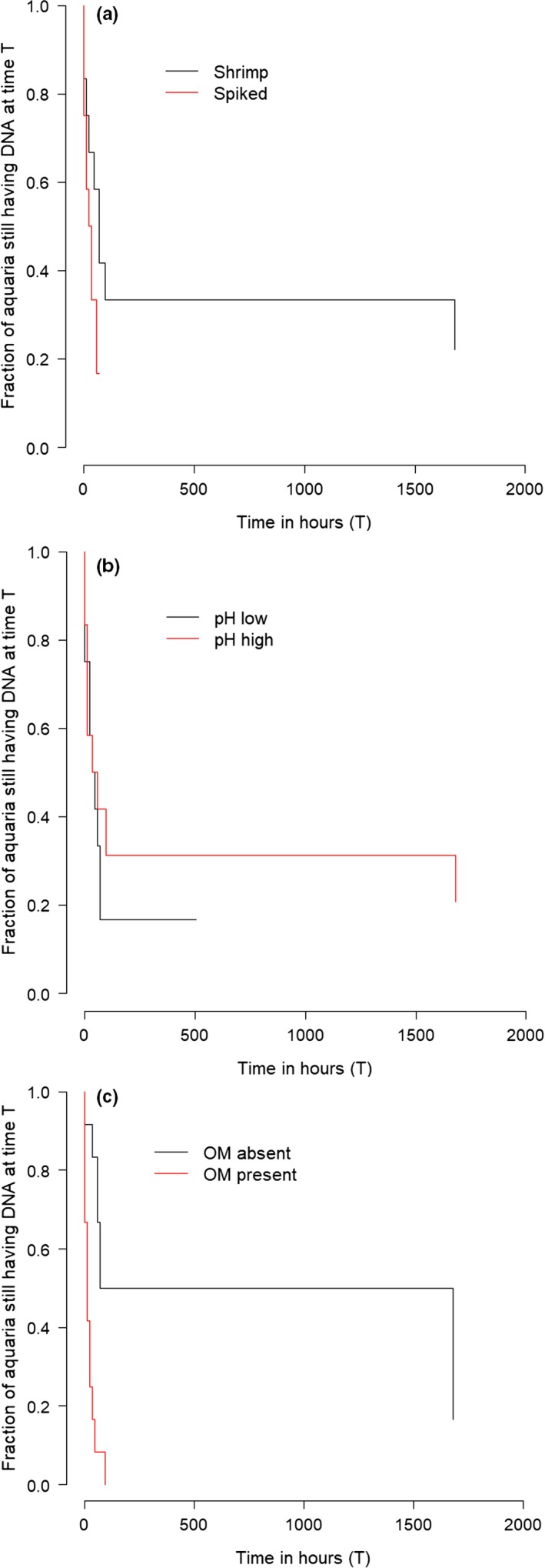
Time in hours until disappearance of eDNA, illustrated as Kaplan–Meier plots. Survival analysis with censored data: (a) stratified on spiked (black, aq. 2, 4, 6, 8, 12, 14, 16, 18, 22, 24, 26 ,28) versus shrimp DNA (red, aq. 1, 3, 5, 7, 11, 13, 15, 17, 21, 23, 25, 27), (b) pH low (black, aq. 1, 3, 4, 6, 11, 13, 14, 16, 21, 23, 24, 26) versus pH high (red, aq. 2, 5, 7, 8, 12, 15, 17, 18, 22, 25, 27, 28), and (c) organic material absent (black, aq. 1, 2, 6, 7, 11, 12, 16, 17, 21, 22, 26, 27) versus present (red, aq. 3, 4, 5, 8, 13, 14, 15, 18, 23, 24, 25, 28)

Significantly less eDNA was accumulated in the aquariums to which additional organic matter was added (*p* < .05, Figures 1 and 2). Ct‐values were on average 5.4 higher in the aquariums to which organic matter was added and on average 1.3 higher in the aquariums with low pH. However, the effect of pH on eDNA accumulation was not significant at the 5% level.

### 3.3 eDNA survival over time

For this analysis, we only used the aquariums that were monitored over time until the eDNA concentration dropped below the limit of detection. The spiked DNA was degraded beyond detectability within 2–60 hr, whereas the eDNA from the live shrimps was degraded in 0–1,680 hr. We performed a survival analysis with the Cox's proportional hazards model to estimate how the treatments affected the time needed for eDNA to degrade beyond the detection limit (45 cycles) (Therneau, 2014; Therneau & Grambsch, 2000). We used OM, pH, and DNA source as treatment groups to estimate the effect of the treatments. eDNA degraded significantly faster in the treatments in which OM was added (*p* = .003), whereas the pH did not significantly affect eDNA degradation (*p* = .360). The survival analysis shows that spiked DNA was degraded significantly faster than the eDNA released by the living shrimps (*p* = .023). The largest difference in eDNA survival versus survival of the spiked DNA was found in the treatment with high pH and no OM. The spiked DNA (treatment F) was degraded between 0 and 12 hr whereas it took between 1,008 and 1,680 hr for the eDNA to degrade (treatment B).

### 3.4 Inhibition

On average inhibition caused qPCRs to be delayed with 1.05 cycle. In 17% of the reactions performed, the qPCR was delayed for more than 2 cycles. We found that qPCRs with 33 template DNA molecules were 3.3 times more often inhibited for more than 2 cycles than qPCRs with 3,333 template DNA molecules. However, inhibition was not significantly stronger at low DNA concentration (slope of regression did not differ from 1 *T* = −0.2081434, *df* = 21, *p* > .4). The model that best supported the data did not include pH or OM as factors. Therefore, we assumed that the low levels of inhibition would affect all treatments equally.

## 4 DISCUSSION

### 4.1 eDNA detection

Prevention of false negatives is an issue that receives much attention in monitoring freshwater biodiversity using environmental DNA (e.g., Buxton, Groombridge, & Griffiths, 2017; Darling & Mahon, 2011). Therefore, a better understanding of the limits of eDNA detection is essential. This study shows that eDNA of live shrimps degrades faster in the presence of OM, resulting in reduced amounts of detectable eDNA, especially when pH is low, as might be found in peat bogs. We found the level of PCR inhibition to be unaffected by pH or the presence of OM. Therefore, detection of reduced amounts of eDNA when OM was present must be explained by a decline in rate of decay and by a failure to sample eDNA instead of by PCR inhibition. As spiked DNA degraded significantly faster than eDNA, we believe most eDNA detected in natural systems must be contained inside cells or mitochondria. This is in line with findings of Turner, Uy, and Everhart (2015) who found that only a minor fraction of carp eDNA to be extracellular. Dupray et al., (1997) report that heat‐killed cells of *Salmonella typhimurium* persist in seawater longer than purified DNA. Nielsen, Johnsen, Bensasson, and Daffonchio (2007) show that the residence time of bacterial DNA in soil is generally longer when dead cells are used as DNA source compared to purified DNA.

In aquatic environments, DNA is known to degrade faster in the presence of DNA‐consuming microorganisms (Alvarez et al., 1996; Dupray et al., 1997). The longer persistence of cellular DNA can be explained by the presence of cellular compounds such as cell membranes that form a barrier against DNA‐consuming microorganisms and nucleases in the environment (Dupray et al., 1997).

Humic acids can strongly adsorb DNA, probably by ligand binding, hydrophobic interaction, aggregation, or precipitation (e.g., Saeki et al., 2011), and eDNA, therefore, might have been adsorbed to organic particles that were deposited at the bottom of the aquariums. Stotzky (2000) found that DNA bound to humic acids and clay‐humic acid complexes becomes more resistant to degradation by DNases. However, as we infer that most eDNA is cellular, these processes might have a minor effect on eDNA contained in cells or mitochondria. Sampling of organic material or sediments might increase the yield of target eDNA, though PCR might be inhibited by organic acids in such cases. However, sampling of organic material or sediments might result in detection of historical eDNA, not representing the actual presence of target species (Olajos et al., 2018).

In a comparable experimental set‐up to ours, using tanks, Buxton et al., (2017) found the effect of pH on eDNA survival to be insignificant (which is in line with our findings), but that sediment has a strong effect. The authors conclude that especially “ponds with organic sediment types—or sediments that become suspended easily—can be a source of false‐negative results” (Buxton et al., 2017). Remarkably, in our aquarium treatment B (high pH and no added OM), eDNA could be detected more than 6 weeks later, whereas other studies found that eDNA degrades beyond detection ability within 2 weeks (Dejean et al., 2011; Eichmiller et al., 2016; Strickler et al., 2015; Thomsen, Kielgast, Iversen, Møller, et al., 2012; Thomsen, Kielgast, Iversen, Wiuf, et al., 2012). However, the eDNA concentrations in these aquariums were unnaturally high, thus not reflecting a natural situation. This might have resulted in relatively high amounts of detectable eDNA and probably lengthened eDNA survival.

Several studies show a correlation between eDNA concentration and population density (Baldigo, Sporn, George, & Ball, 2017; Maruyama et al., 2014; Wilcox et al., 2016). This study, as well as previous studies (Eichmiller et al., 2016; Strickler et al., 2015) show that environmental conditions strongly affect eDNA concentration. We, therefore, believe caution is warranted when using eDNA concentrations as proxy for population density. Environmental conditions might specifically affect eDNA concentrations on the sampling site. Therefore, it is necessary to correct measured eDNA concentrations for local environmental conditions such as pH and amount of OM.

Our study, as well as previous studies, focused on selected environmental factors only and was conducted in an artificial ecosystem (i.e., an aquarium; Nielsen et al., 2007). Complex interactions between eDNA degradation and additional factors such as the presence of DNA‐consuming microorganisms remain largely unknown, and future studies should, therefore, include microbial activity as well. In addition, species that occur in a wide range of habitats should be used to investigate the relation between amount of detectable eDNA and other environmental conditions in the field such as seasonality (de Souza, Godwin, Renshaw, & Larson, 2016) or soil type (Buxton et al., 2017).

## CONFLICT OF INTEREST

None declared.

## AUTHOR CONTRIBUTIONS

KB, BG, FTB, and KKB designed the experiments. KB and KKB performed the experiments. LH, KB, and RAV analyzed the data. KB wrote the first draft of the manuscript and all authors revised the manuscript, KB, LH, and FTB finalized the manuscript.

## Data Availability

Data have been deposited at: https://figshare.com/s/67a918dd1a2983fd0a6e and https://figshare.com/s/d798dd569a801802eec2
