# Supplementary figures and images for: Organic matter reduces the amount of detectable environmental DNA in freshwater
Source: Ecol Evol. 2020 Mar 21;10(8):3647–54. doi: 10.1002/ece3.6123 (PMC7160167; doi:10.1002/ece3.6123)

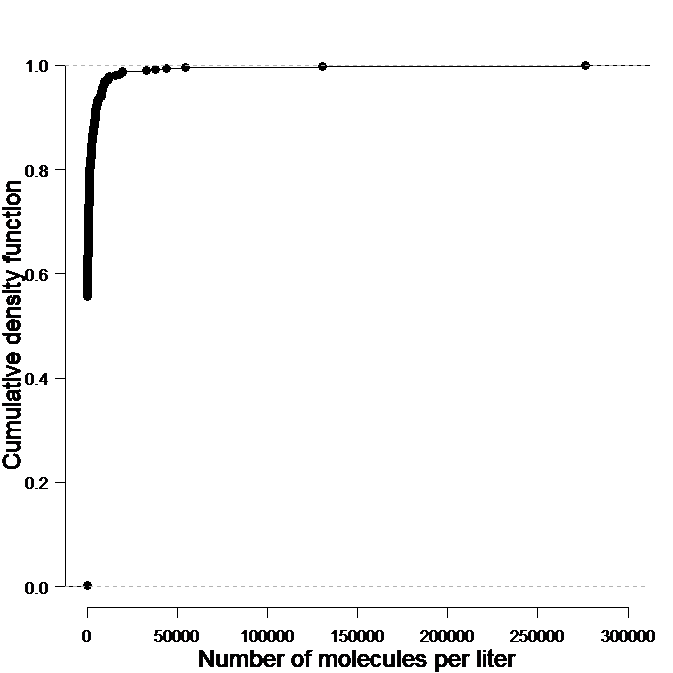

Supplement: Supplementary file 1 [file ECE3-10-3647-s001.tif]

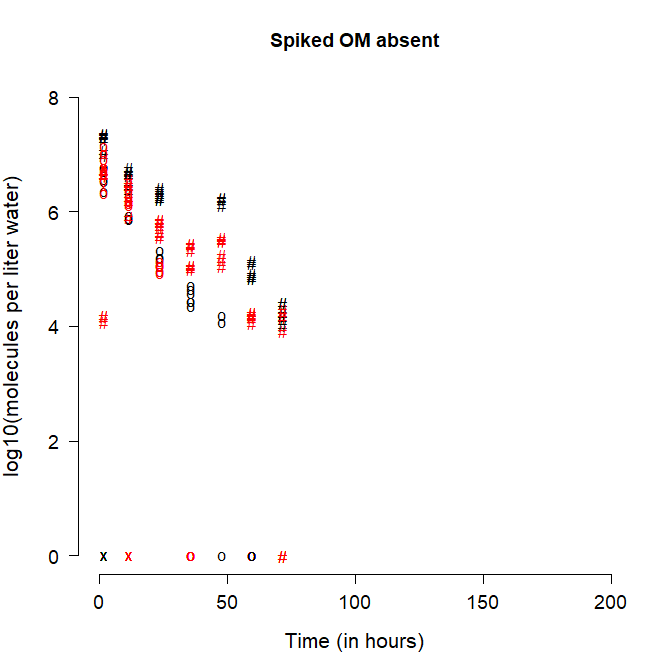

Supplement: Supplementary file 2 [file ECE3-10-3647-s002.tif]

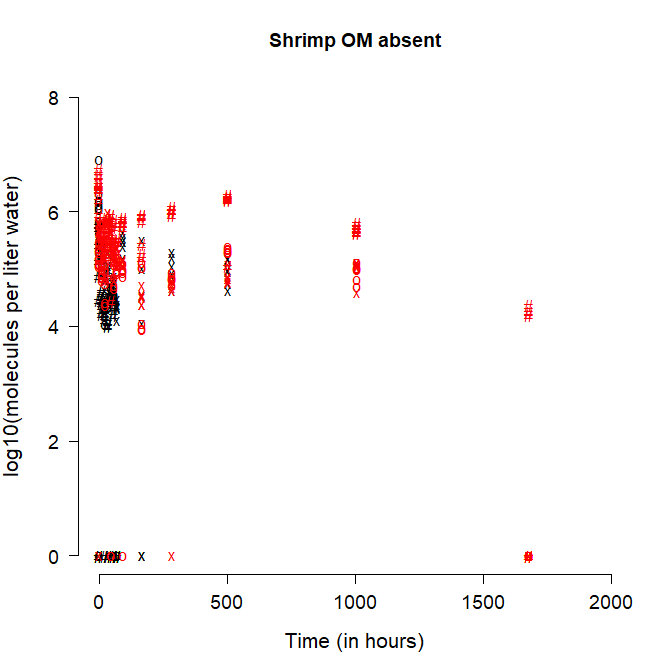

Supplement: Supplementary file 3 [file ECE3-10-3647-s003.tif]

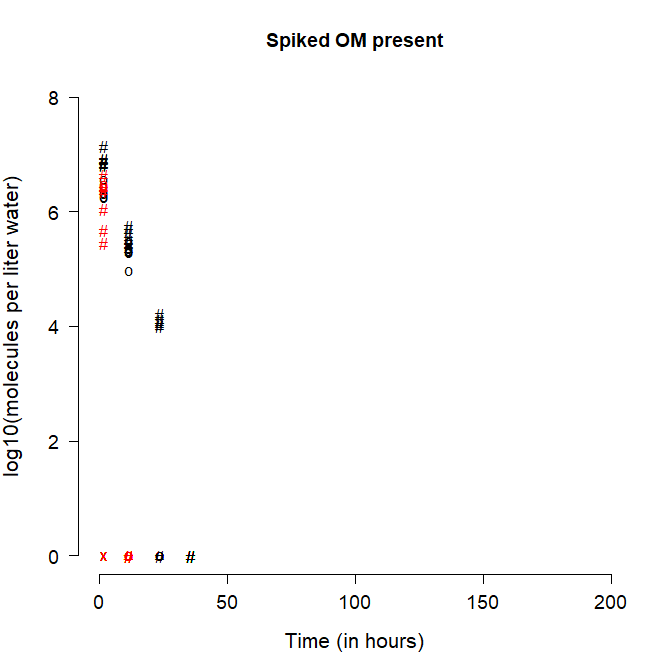

Supplement: Supplementary file 4 [file ECE3-10-3647-s004.tif]

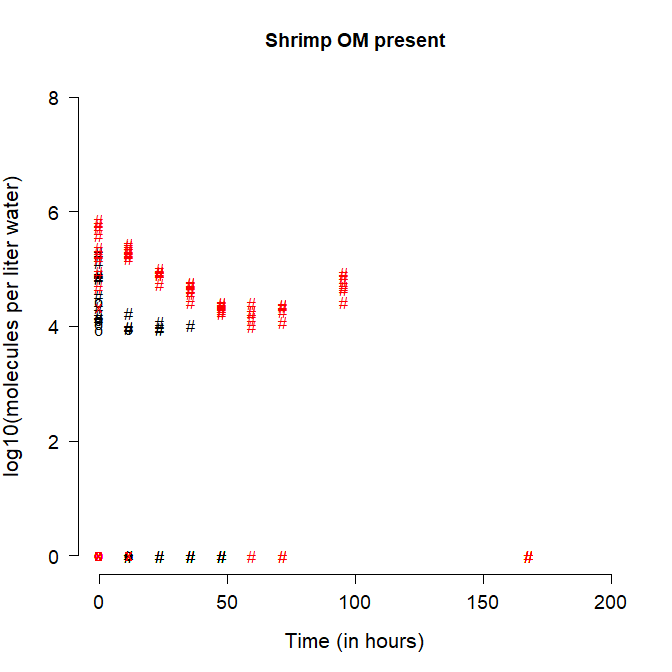

Supplement: Supplementary file 5 [file ECE3-10-3647-s005.tif]
